# Supplementary material for: Content-rich biological network constructed by mining PubMed abstracts
Source: BMC Bioinformatics. 2004 Oct 8;5:147. doi: 10.1186/1471-2105-5-147 (PMC528731; doi:10.1186/1471-2105-5-147)
Supplement: Additional File 2 — The original results of the above study (non-essential files are deleted to keep the file size under the limit set by BMC bioinformatics). [file 1471-2105-5-147-S2.bz2 › chilibotAdditionalFile2/dip40/554ID10049744E10914/html/IFNAR1_IFNB1.html]

 


 **IFNAR1** and **IFNB1** 
  
Found 357 abstracts in PubMed, retrieved 40.  
 

 What does Google say? 
 PDF only 
| .edu only 

---

**Interactive relationship** (e.g. stimulation, inhibition, etc)

**Inhibitory relationship**- However, two mAb that bound to sites remote from the IFNAR  [ **IFNAR1** ]  2 binding site on IFN beta  [ **IFNB1** ]  also inhibited IFN beta  [ **IFNB1** ]  IFNAR  [ **IFNAR1** ]  2 complex formation and demonstrated potent neutralizing activity.  Ref: 11747625 J Interferon Cytokine Res, 2001
- Cell surface levels of IFNAR 1  [ **IFNAR1** ]  decreased and Stat1 levels increased after IFN beta  [ **IFNB1** ]  treatment, and retreatment with IFN resulted in an attenuated induction of Mx protein expression.  Ref: 12034032 J Interferon Cytokine Res, 2002
- Interferon alpha  [ **IFNAR1** ]  effectively inhibited SARS CoV replication, but with a selectivity index 50 90 times lower than that for interferon beta  [ **IFNB1** ] .  Ref: 12892961 Lancet, 2003
**Neutral relationship**- In murine macrophages, IFN beta  [ **IFNB1** ]  induced TLR3 up regulation required  **IFNAR1** , STAT1, and in part IRF 1, but not the Janus kinase Jak family member Tyk2.  Ref: 12672806 J Biol Chem< MedlineTA>J Biol Chem, 2003
- The higher antiviral effect of IFN beta  [ **IFNB1** ]  was observed when the interferon alpha  [ **IFNAR1** ]  beta receptor IFNAR  [ **IFNAR1** ]  expression in the liver increased, and the lower effect was observed when its expression decreased.  Ref: 12501006 Jpn J Pharmacol, 2002
- The time dependent expression of IFNAR  [ **IFNAR1** ]  was supported by that of transcription factor level induced by interferon beta  [ **IFNB1** ] .  Ref: 11388696 Life Sci, 2001
- Interferon alpha  [ **IFNAR1** ]  is associated with a greater delay in gastric emptying and a higher symptom score than is interferon beta  [ **IFNB1** ] .  Ref: 11837736 Dig Dis Sci, 2002
- These results suggest that selecting the most suitable dosing time of IFN beta  [ **IFNB1** ] , associated with the 24 h rhythm of IFNAR  [ **IFNAR1** ]  expression in the liver, may be important to increase effectively the antiviral activity of the drug in experimental and clinical situations.  Ref: 12501006 Jpn J Pharmacol, 2002

**Non-interactive relationship** (e.g. studied together, co-existance, homology, etc.)

- Two of these inhibited IFN beta  [ **IFNB1** ]  IFNAR  [ **IFNAR1** ]  2 complex formation, suggesting that steric hindrance of receptor binding constitutes their mechanism of neutralization.  Ref: 11747625 J Interferon Cytokine Res, 2001
- Moreover, it is not clear whether there are any differences among interferon alpha  [ **IFNAR1** ] , interferon beta  [ **IFNB1** ] , and interferon gamma in the treatment of liver fibrosis.  Ref: 11940252 BMC Cell Biol, 2002
- To investigate the intra hepatic activation of the IFN system in patients affected by chronic HCV infection in comparison with that observed in a non infectious liver disease such as non alcoholic steatohepatitis, we measured the liver steady state mRNA levels of interferon alpha  [ **IFNAR1** ] , interferon beta  [ **IFNB1** ]  and interferon gamma as well as of IFN related genes IFNAR 1  [ **IFNAR1** ] , STAT1alpha, PKR, 2 5 AS, IRF 1, ICE and IL 18.  Ref: 12794720 J Med Virol< MedlineTA>J Med Virol, 2002
- Both rat interferon beta  [ **IFNB1** ]  and interferon gamma reduced smooth muscle alpha actin abundance after 6 days treatment, but rat interferon alpha  [ **IFNAR1** ]  did not alter smooth muscle alpha actin level.  Ref: 11940252 BMC Cell Biol, 2002
- Considering the increased risk of reactivation of autoimmune phenomena during a six months therapy with interferon alpha  [ **IFNAR1** ] , an intensive combination therapy with interferon beta  [ **IFNB1** ]  and gamma 2 weeks 1x3 MIU nIFN beta Fiblaferon R i.  Ref: 12772056 Z Gastroenterol< MedlineTA>Z Gastroenterol, 2002
- RESULTS Rat interferon beta  [ **IFNB1** ]  and interferon gamma significantly inhibited rat hepatic stellate cell proliferation while rat interferon alpha  [ **IFNAR1** ]  did not affect the cell proliferation under the same culture condition.  Ref: 11940252 BMC Cell Biol, 2002
- Ibuprofen, iloprost, imatinib mesilate, imiquimod, interferon alpha  [ **IFNAR1** ]  2b, interferon alpha  [ **IFNAR1** ]  2c, interferon beta  [ **IFNB1** ] .  Ref: 12087878 Methods Find Exp Clin Pharmacol, 2002
- Other pro inflammatory cytokines representing potential therapeutic targets include interferon beta  [ **IFNB1** ] , interferon alpha  [ **IFNAR1** ] , IL 6, IL 15, IL 17 and IL 18.  Ref: 12769749 Curr Pharm Des, 2003
- CONCLUSIONS Our results indicate that rat interferon alpha  [ **IFNAR1** ]  and interferon beta  [ **IFNB1** ]  have different biological effects on rat hepatic stellate cells and suggest that there are different signaling events between interferon alpha  [ **IFNAR1** ]  and interferon beta  [ **IFNB1** ]  in hepatic stellate cells.  Ref: 11940252 BMC Cell Biol, 2002
- Another subset of dendritic cells in humans is the plasmacytoid dendritic cell in peripheral blood, which is characterized by the production of large amounts of type I interferon interferon alpha  [ **IFNAR1** ]  and interferon beta  [ **IFNB1** ]  upon viral infection.  Ref: 12445198 J Invest Dermatol, 2002
- Relationship Between 24 Hour Rhythm in Antiviral Effect of Interferon beta  [ **IFNB1** ]  and Interferon alpha  [ **IFNAR1** ]  beta Receptor Expression in Mice.  Ref: 12501006 Jpn J Pharmacol, 2002
- Lymphocytic choriomeningitis virus LCMV induces type I interferon alpha  [ **IFNAR1** ]  and beta interferon  [ **IFNB1** ]  IFN alpha and IFN beta  [ **IFNB1** ]  upon infection and yet is sensitive to the addition of type II interferon gamma interferon IFN gamma to the culture media.  Ref: 11390623 J Virol, 2001
- We analyzed whether interferon alpha  [ **IFNAR1** ]  2b IFN alpha 2b and IFN beta  [ **IFNB1** ]  1a engage their common receptor to generate activated receptor complexes possessing distinct signaling properties.  Ref: 11911800 J Interferon Cytokine Res, 2002
- Strong inhibition of Ewing tumor xenograft growth by combination of human interferon alpha  [ **IFNAR1** ]  or interferon beta  [ **IFNB1** ]  with ifosfamide.  Ref: 12400012 Oncogene, 2002
- Therefore, our objective in current study is to investigate the effects of rat interferon alpha  [ **IFNAR1** ] , interferon beta  [ **IFNB1** ] , and interferon gamma on the proliferation and activation of rat hepatic stellate cells.  Ref: 11940252 BMC Cell Biol, 2002
- the 1B 2R1 replicon was sensitive to interferon alpha  [ **IFNAR1** ] , interferon beta  [ **IFNB1** ] , and interferon gamma.  Ref: 12810084 Biochem Biophys Res Commun, 2003
- ... interferon alpha  [ **IFNAR1** ]  IFN alpha and IFN beta  [ **IFNB1** ]  were found to suppress the transcription driven from both promoters.  Ref: 12201906 AIDS Res Hum Retroviruses, 2002
- Interferon alpha  [ **IFNAR1** ]  and Interferon beta  [ **IFNB1** ]  can inhibit recurrence in HCC patients with HCV infection background, though the mechanism is not fully understood.  Ref: 12679900 World J Gastroenterol, 2003
- Thyroid dysfunction and autoimmunity have been reported during type I interferon therapy, namely interferon alpha  [ **IFNAR1** ]  for chronic hepatitis or interferon beta  [ **IFNB1** ]  for multiple sclerosis.  Ref: 11502774 J Clin Endocrinol Metab, 2001
- Interleukin 1beta and interleukin 1 receptor antagonist levels were measured in 15 patients who had a recent diagnosis of subacute sclerosing panencephalitis group patients who had been treated with isoprinosine group patients with intraventricular interferon alpha  [ **IFNAR1** ]  group 3, and 6 patients with interferon beta  [ **IFNB1** ]  group 4.  Ref: 11417607 J Child Neurol, 2001
